# MicroGal Gravity Measurements with MGS-6 Micro-g LaCoste Gravimeter

**DOI:** 10.3390/s19112592

**Published:** 2019-06-06

**Authors:** Marek Przyborski, Jerzy Pyrchla, Krzysztof Pyrchla, Jakub Szulwic

**Affiliations:** 1 Department of Geodesy, Gdansk University of Technology, 80-233 Gdańsk, Poland; marek.przyborski@pg.edu.pl (M.P.); jerzy.pyrchla@pg.edu.pl (J.P.); 2 Faculty of Electronics, Telecommunications and Informatics, Gdansk University of Technology, 80-233 Gdańsk, Poland; krzpyrch@student.pg.gda.pl

**Keywords:** relative gravimetry, marine gravimeter, tides measurements, drift of gravimeter

## Abstract

The article discusses the registration of micro-gravity changes with the MGS-6 Micro-g LaCoste gravity sensor during static measurements. An experiment was carried out to determine how small changes in gravity can be registered using the MGS-6 system sensor. The tides of the Earth’s crust were chosen as the source of disturbance of the field with small amplitude and long-term changes. The tested sensor was placed in a geophysical observatory on a specially designed tripod. Simultaneously on the nearby concrete pillar, the registration of changes in gravity was carried out using the superconducting iGrav gravimeter. The high temporal stability of the superconducting gravimeters and the low noise combined with leading sensitivity of its reading allow it to be considered as a reliable reference source for MGS-6. The article discusses the impact of non-leveling changes of the MGS-6 gravimetry on the reading and determines the size of its non-linear drift. The obtained differences in indications between devices did not exceed 50 μ Gal for 68% of data. The measurements also showed excellent time stability of the MGS-6 measurement system. The data collected during the experiment allowed determining the level of accuracy that can be sought during real measurements using the MGS-6 system on research vessels. They also give an overview of the dynamics of the drift phenomenon of the analyzed research system.

## 1. Introduction

The measurement of the Earth’s gravitational field provides essential data for solving problems in many scientific fields [1]. The mobile gravimetry with the development of the Global Navigation Satellite System (GNSS) technique has become an excellent way to determine the high resolution local gravity field of Earth in various scenarios, such as airborne and land or sea based moving platforms [2,3]. In recent years, many constructions of relative gravimeters have been developed, considered to be mobile. However, this term does not necessarily mean that the given gravimeter is adapted to work on a mobile platform. The word mobile is even used to describe gravimeters whose transfer is a complex procedure and the place of their installation must meet many requirements [4]. According to this principle, we adopt a more strict division into gravimeters requiring installation on a solid foundation and those that can perform measurements while being installed on mobile platforms. Measurements performed by a gravimeter installed on a mobile platform (sea and air) are much more noisy with environmental stimuli than made with a gravimeter located on a solid ground [5,6]. They are the most effective technique of providing accurate data with the high spatial resolution of 20 m or better [7]. The continuous development of gravimetric systems such as ZLS Dynamic Meter, Micro-g LaCoste MGS-6, Elektropribor Chekan, and Canadian Micro Gravity AM GT-2M provides a great demand for this type of measurements [8,9,10]. They are used in areas that do not have good coverage, and the surface is unstable or covered with water. It is an ideal technique for supplementing data obtained from current satellite missions and being the basis for many applications of the regional gravitational field model. Gravimetric measurements made on mobile platforms can be used to check the validity of existing data sets. They are also an effective tool providing imaging of the gravimetric field in the areas connecting sea with land-based ones. The gravimeter is moving while recording measurements. Due to the need to maintain the sensor during horizontal and vertical measurements, it is mounted on a mechanically stabilized platform. In MGS-6 gravity sensor, sensor stabilization is achieved in several stages. Frame supports stabilizer (Gimbal) and sensor and isolates it from vibrations using suspension cables (Suspension Cords), air dampers (Air Dampers) and pneumatic vibration mounts (Air-Filled Vibration Mounts). Computer software is the support for the above-mentioned system [11]. Optimization of stabilized reference parameters exceeds the solutions used in the first constructions of this type of gravimeters [12,13]. The gravity meter is equipped with an advanced data collection system, synchronized with the rubidium clock. The clock can be used to synchronize the measurements of other sensors installed on the mobile platform. An important issue is the determination of the accuracy of the registration of gravimetric measurements in the absence of disturbances caused by man [14], it allows to check the accuracy of registration and the scale factor [15,16]. Achieving such a goal required the team planning measurements to perform test measurements taking into account data on local hydrology [17]. When choosing a location for observation, a place was chosen where gravimetric measurements were conducted for several decades and the influence of hydrological changes is known. Bearing in mind this type of disorder, and taking into account the data from longtime registrations the mount July was selected for measurements. According to [18], the changes related to hydrology in this month should have a negligible impact on the sensor. Such classical processing is briefly presented for which gravity variations due to hydrology can be found in the literature [19].

The team also modeled the atmospheric contribution to gravitational changes, including atmospheric pressure micropulsing [20]. The atmospheric influence was modeled using a simple, linear model described in [21]. The meteorological station of the laboratory collected the barometric data used for this estimation. The influence of this effect is nearly negligible because its maximal magnitude was 3 microgals. During the preparation and execution of the measurements, the most important effect was to determine the sensitivity level of the device. The obtained result was decided to be treated as the asymptote of this parameter during real measurements, the value to which a well-filtered signal will strive. The article describes the conditions of the measurements carried out. The results recorded by the gravimeters participating in the study are presented. By performing initial data filtration, broadband seismic noise [22] was removed so that they would not affect the result of the sensitivity analysis of the gravimetric system. The analysis of the acquired data was used to develop assumptions for field measurements. They form the basis for the development of mathematical tools supporting filtering and correction of data obtained during offshore measurement sessions. All these activities were aimed at optimizing the use of the ship and the gravimetry as the basic measurement equipment. The measurements were recorded at the research-pattern gravimetric laboratory in the geophysical observatory Borowa Gora (Poland) and concern the MGS-6 gravimetric sensor.

## 2. Materials and Methods

The research team transported the MGS-6 system sensor, its UPS (PG800-UPS by AJ’s Power Source, Inc, 6931 Land O Lakes Blvd. Land O Lakes, FL 34638, USA), a timing unit and Power Electronic Box to the research-pattern gravimetric laboratory Borowa Góra. The UPS provided the appropriate level and stability of the supply voltage for the measuring electronics of the device. The temperature of the sensor before transport was maintained at 55.8 ${}^{\circ }C$ for more than two weeks, so the manufacturer’s requirements regarding the drift stability conditions of the device were met. During transport, the device was heated continuously using the Auxiliary Heater Box connected to a Dc-Ac converter powered by a gel battery.

**Figure 1:**
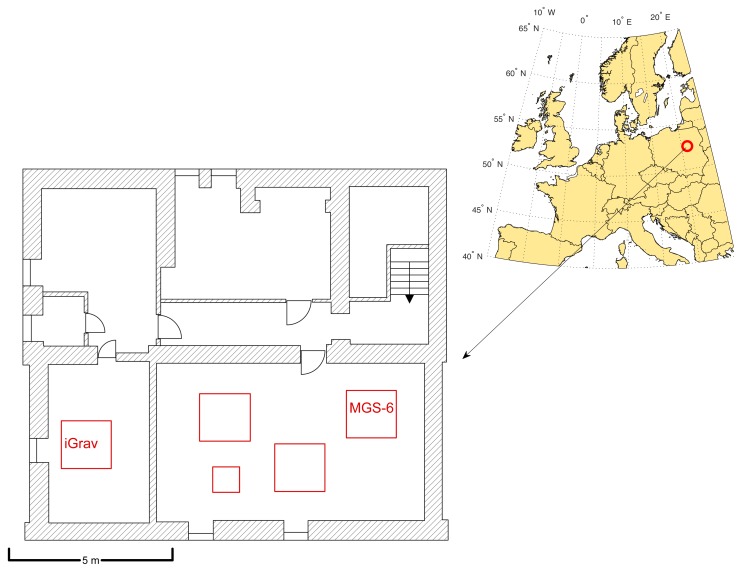
The map presents the location of measurements and a detailed plan of the laboratory. Red squares indicates the gravimetric pillars.

Data registration using MGS-6 and iGrav gravimeters was performed in the research-pattern gravimetric laboratory in the geophysical observatory Borowa Góra (Figure 1). The observatory is subordinate to the Institute of Geodesy and Cartography located in Warsaw. The device was placed on a specially designed concrete pillar measuring (1.2 × 1.2 × 1.5) m separated from the foundations of the building. The measurement pillars of the laboratory are located underground and are separated from buildings fundaments to provide isolation from disturbing vibrations.

Data registration started at (UTC) 14:16:19 on 5 July 2018 and ran until 14:50:52 on 19 July 2018. At the time of registration, the gravimetric sensor was placed on a tripod used to set ground gravimeters. It allowed leveling the device without the use of an active gimbal. After setting the sensor on a tripod and connecting with the target electronics, the sensor leveling was performed. Leveling was made based on the indications of the tylatometers integrated with the sensor. The operation was carried out until the horizontal acceleration indications lower than 1 mGal for each axis were obtained. To reduce the effect resulting from settling the tripod screws after 12 h, the device was re-leveled. After the second leveling, the laboratory was closed and no other interference in the work of the sensor was done.

During measurements, the electronic gimbal was not chosen for several reasons. First, the measurements were made in an isolated environment from any significant inclinations. Secondly, it was considered that the corrections introduced by gimbal due to the low amplitude of inclinations may introduce larger disturbances to the result than the heel itself and calculating their impact would be complicated. Thirdly, it was assumed that the laboratory could go through a seismic wave which registration could be very interesting. Disorders resulting from gimbal movements could be associated with the recorded wave and make its detection difficult. Thus, in the experiment, gimbal was seen as a potential source of high-frequency interference.

Superconducting gravimeters are characterized by almost zero drift, very low own noise and excellent linearity in the range of microglial acceleration changes [23]. For the reason of its stability, it is well suited as a benchmark for testing much less accurate relative gravimeters.

## 3. Results

The first 48-h and last 15 min of data recorded during measurement session were removed before the analysis. Such choice was dictated by the desire to minimize the potential impact of the process of the stopover on the measurement data. Initial data were removed because the fact that during the first 24 h the gravimeter was still leveled and in real situations the actual measurements did not start faster than 24 h after the sensor was re-calibrated.

**Figure 2:**
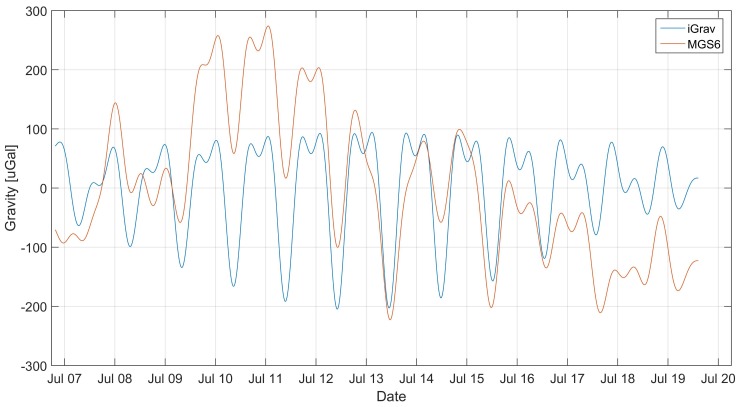
Filtered signal from MGS-6 gravimeter superimposed on the filtered signal from the gravimeter iGrav.

The data were checked for pins and registration breaks. Due to the shortness of the session, none of these phenomena were observed. The fixed data were removed from the collected data by subtracting the mean value of the waveform. The data were then filtered using a Butterworth IIR filter of 8’th order, with a cutoff frequency of 30 μHz (2.592 CyclesPerDay). The filtration was subjected to both data from the superconducting gravimeter and the MGS-6 gravimeter. Forward-Backward [24] method was used for filtration to eliminate the effect of phase shift. The data obtained in this way were superimposed on each other, as shown in the Figure 2.

**Figure 3:**
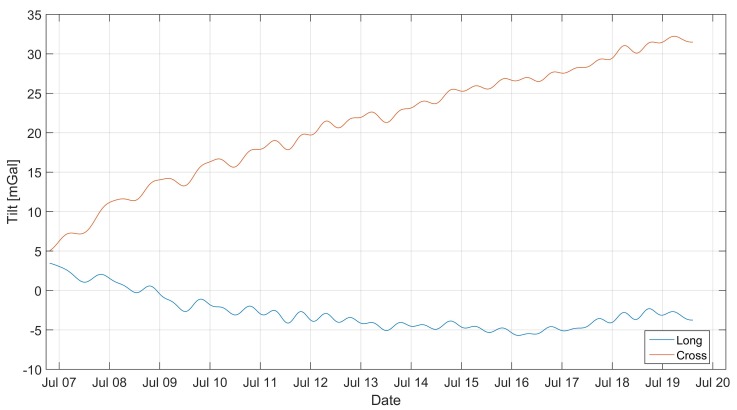
Changes in the gravimetric tilt expressed as the value of the acceleration of the perpendicular to the axis of the sensor. Measured using built-in tiltmeters

The indications of the tiltmeters shown in Figure 3 show that the gravimeter tilted during the experiment. In order to eliminate the influence of tilt on the long-term variability of the gravimeter’s indications, the tilt correction was introduced. It was assumed that the deflections of the sensor’s axis from the vertical line were small and slow-changing, so you can neglect the effects related to the gravimetric movement with respect to the measuring plate. The sensor software calculated, in real time, the acceleration value on axes perpendicular to the axis of the instrument. Knowing at the moment the value of perpendicular accelerations $a_{L}$ and $a_{C}$ and the grated gravity $g_{r}$ we could calculate the gravity vector module as:

(1) $$g=\sqrt{g_{r}^{2}+a_{L}^{2}+a_{C}^{2}}$$

Accepting the following $a_{L}^{2}+a_{C}^{2}=a^{2}$, we develop the equation in the Maclaurin series. With accuracy to the terms of the sixth row, the development is shown by the equation:

(2) $$g=g_{r}\left(1+\frac{a^{2}}{2g_{r}^{2}}-\frac{a^{4}}{8g_{r}^{4}}+\frac{a^{6}}{16g_{r}^{6}}+\ldots \right)$$

**Figure 4:**
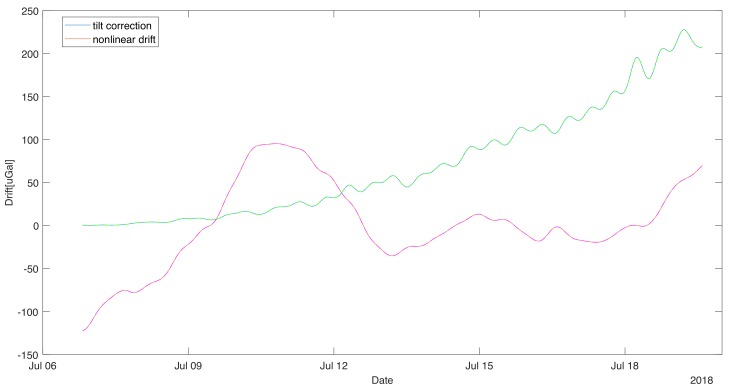
Tilt correction value and long-term variability of MGS-6 gravimetric indication (residual). The long term variability was accepted as an estimation of non-linear drift. Dashed lines indicate the approximation errors.

In the situation of smaller tilts, it is possible to neglect all term despite first non-zero. In that situation, the tilt correction will be a quadratic function of the horizontal acceleration [25]. In our case, this assumption is correct during the experiment. We decided to calculate the tilt correction to the term of second order and treat the next non-zero term (of the fourth order) as the approximation error estimate. Figure 4 shows the variation of the tilt correction and its estimate of error as dashed lines. The error introduced by neglecting higher order terms is much smaller than one uGal, so the lines nearly overlap.

The removal of the influence of tilt on the recorded data makes possible the analysis of slow-changing components. Figure 4 shows a graph of the slow-changing component obtained by filtering the measurement data corrected by the tilt with the moving-average filter with the window length equal to 48 h. This allowed for the evolution of changes in the sensor readings whose main cause is nonlinear drift [26]. It can be easily seen that the tilt correction is in the same order as the drift. If it was not calculated, it could be easily interpreted as the linear component of the sensor drift.

**Figure 5:**
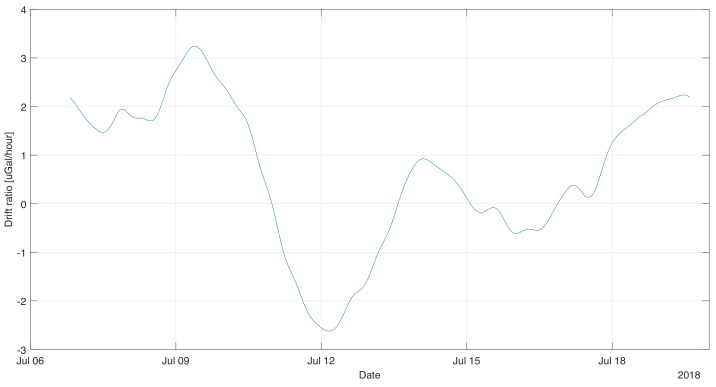
Instantaneous value of the gravity drift calculated on the basis of its estimate.

Based on the calculated long-term variability of the gravimetric indications, the instantaneous drift of the device was estimated. It was calculated as a time derivative of the data presented in graph Figure 5. The obtained data was again filtered with an averaging filter (window 24 h) in order to remove the artifacts created during numerical differentiation. It should be noted that the drift value changes significantly during the measurement. Its maximum value was 2.32 mGal/month, however, along with the duration of the measurement there was a decrease. After 9 days of instantaneous registration, drift values did not exceed 1.6 mGal/month. The average drift value was 1.4 mGal/month during the measurement. However, it should be noted that the device tends to drift more than a few days after installation, therefore it can be assumed that it is possible to achieve a smaller average drift in the case of long static sessions.

**Figure 6:**
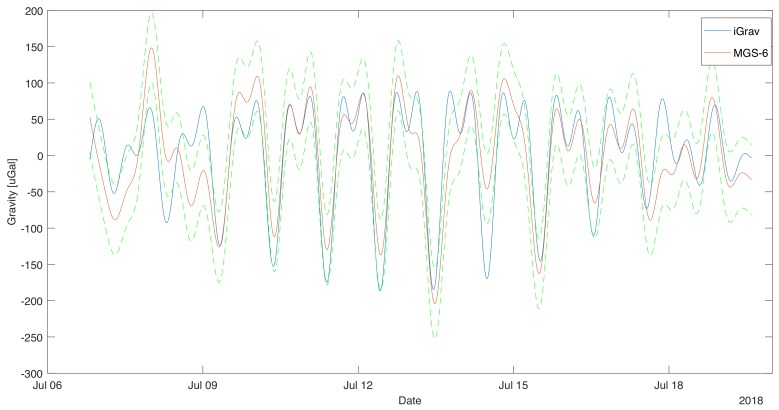
Signal from gravimeter MGS-6 superimposed on the signal from the gravimeter iGrav. Rollover correction is included here. The nonlinear drift was subtracted. Dashed lines indicate the uncertainty estimate for MGS-6.

**Figure 7:**
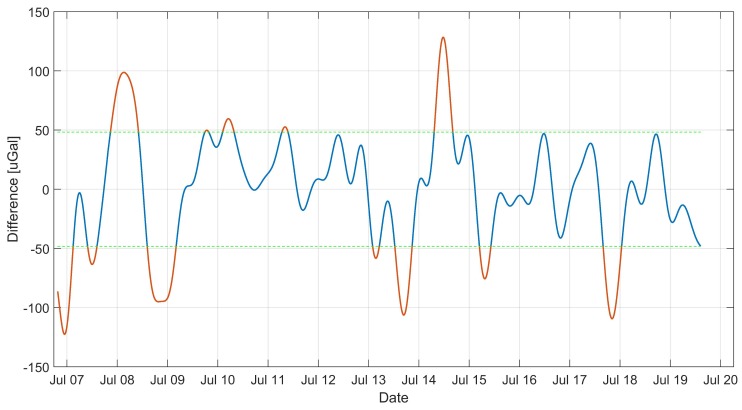
Difference between the MGS-6 and iGrav gravity indications. The lines indicate a 1σ range.

**Figure 8:**
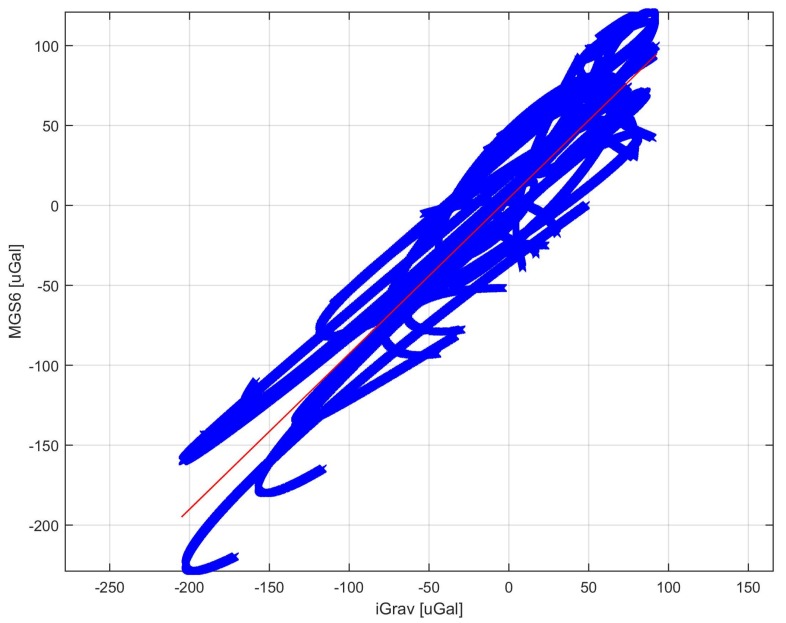
Graph of gravity MGS-6 indication dependence on the iGrav gravimetric indication.

In order to analyze the tidal data from the filtered and adjusted by the heeling of the record, the long-term component was subtracted, this resulted in the removal of the drift from the results. The result obtained is presented in Figure 6. The difference between the gravimetric value of iGrav and MGS-6 was calculated. The result is shown in Figure 7. The average value of the difference was close to zero of −2 μGal. This means that MGS-6 gravity drift has been well estimated and there is no significant discrepancy in mileage. The standard deviation of the difference in readings is presented in diagrams Figure 7 using dashed horizontal lines. It is 48 μGal, it was taken as the limit uncertainty of measurement for MGS-6. It was also checked whether there is a difference in gravity ratios of MGS-6 and iGrav. To this end, data were used for which the difference between sensor readings was less than 1σ. This caused rejection of approx. 27% of the data. The data selected in this way are presented in the graph of MGS-6 indication dependence on iGrav. A straight line was fitted to the data. The result is shown in Figure 8. The fit ratio $R^{2}$ was 0.896. The directional coefficient of the line has been defined as 0.97 means that the potential difference in the scale factors of both gravimeters is less than 3%.

## 4. Conclusions

The fast development of gravimeters installed on mobile platforms started from the 1950s to the present day is due to the analysis of their work in various conditions [10,24]. For this reason, sensors of this type have been optimized for the ability to work in difficult conditions. Therefore, it is not surprising that parameters such as the dynamic range are particularly large in this type of devices. In this situation, it should be expected that the sensitivity of the device may be smaller than in constructions built in similar technology but designed to work in better conditions. Therefore, the study focused on determining the level of measurement accuracy that a mobile sensor can achieve. According to experimental data, it was found that the device is able to register acceleration changes greater than 50 μGal. In the range of accelerations recorded by the device, the response of the device was linear. The aim of the study was to determine the limit sensitivity of the device. For this reason, during the experiment, the influence of all disturbing factors was limited, making the basic source of noise in the reading the own noise of the device. Experimental team decided to determine the duration of the experiment for two weeks. It was tried to make the measurement time as close as possible to the duration of the marine measurement campaigns. This allowed stating that the device, even in such a short measuring campaign, can perform detection at a much lower level than it is done during marine gravity measurements. In addition, a short measuring period meant that no changes in gravity were registered that would be associated with seasonal effects, such as described in [27]. The measurement showed how important time is from the installation of the device on the ship until the start of the measurement trip, due to the drift of the device. Conducted research suggests that it is possible to take measurements with accuracy to tens of μGal as long as noise and interference are properly filtered. The team concluded that it will be very interesting to conduct a longer study using an artificial acceleration source of a known nature. This would allow determining both the impulse response of the device and the exact check of the scale factor of the sensor. Measurements based on tides can not suffice for determining this parameter with the accuracy given in [28]. This is due to the fact that the determined uncertainty of the sensor measurement is only several times lower than the tidal amplitude.
